# Modelling human mobility patterns using photographic data shared online

**DOI:** 10.1098/rsos.150046

**Published:** 2015-08-12

**Authors:** Daniele Barchiesi, Tobias Preis, Steven Bishop, Helen Susannah Moat

**Affiliations:** 1 Department of Mathematics, University College London, Gower Street, London WC1E 6BT, UK; 2 Warwick Business School, University of Warwick, Scarman Road, Coventry CV4 7AL, UK; 3 Department of Physics, Boston University, 590 Commonwealth Avenue, Boston, MA 02215, USA

**Keywords:** computational social science, data science, social media, *Flickr*, human mobility, complex systems

## Abstract

Humans are inherently mobile creatures. The way we move around our environment has consequences for a wide range of problems, including the design of efficient transportation systems and the planning of urban areas. Here, we gather data about the position in space and time of about 16 000 individuals who uploaded geo-tagged images from locations within the UK to the *Flickr* photo-sharing website. Inspired by the theory of Lévy flights, which has previously been used to describe the statistical properties of human mobility, we design a machine learning algorithm to infer the probability of finding people in geographical locations and the probability of movement between pairs of locations. Our findings are in general agreement with official figures in the UK and on travel flows between pairs of major cities, suggesting that online data sources may be used to quantify and model large-scale human mobility patterns.

## 1. Introduction

Human mobility is governed by individuals' decisions, habits and life experiences. Yet, whenever analysed across large segments of the population, movements exhibit surprising statistical regularities that have been studied in contexts as diverse as anthropology [1], urban mobility and planning [2–4], crime modelling [5], advertising [6] and epidemic spread [7–9].

Recent years have witnessed a rapid increase in the adoption of new communication channels such as mobile phones and online social media. This has opened up an opportunity to create improved models of human mobility, which allow us to better understand the influence of social networks on how people move around [10,11], and even generate predictions on where people might move next [12,13]. New data sources have enabled research about human mobility and, more generally, about human behaviour [14–29].

Previous work drawing on data from mobile phone logs and recorded movements of bank notes has suggested that the travel lengths of journeys people make follows a power-law distribution [30,31]. This corresponds to a behaviour whereby a large number of small movements are occasionally followed by very large ones, a property associated with so-called Lévy flights. Here, we start from this observation, and draw on data from the photo-sharing website *Flickr*, to propose a model of mobility where displacements are grouped together into geographical clusters. We argue that these clusters correspond to latent states or ‘contexts’ that drive the emergence of Lévy flights. Whereas previous work in this vein has been limited to the analysis of the movements of one individual [32], our model is based on the observation of trajectories of 16 000 photographers, and allows us to infer general patterns from the behaviour of many individuals.

## 2. Results

### 2.1 Individuals' mobility model

The location of individuals who posted photos on *Flickr* displays a Lévy-flight pattern, whereby local movements around a relatively small area are occasionally followed by larger movements to a distant area (electronic supplementary material, figure S1). We propose that this behaviour can be modelled by clustering the geo-tagged information for each user into local groups of photos taken in distinct geographical areas, and by studying the statistical properties of sequences of photos within and between clusters.

To cluster groups of local geo-tagged pictures, we employ DBSCAN [33], a clustering technique used to identify an unspecified number of clusters of arbitrary shape. This method is suited to the problem considered here, where we do not have prior information about the number or shape of clusters. Using DBSCAN, two points *p*_1_ and *p*_2_ are regarded as belonging to the same cluster if there is a sequence of intermediate points {*p*_*n*_} that connect them, such that every intermediate point *p*_*n*_ contains *M* other points within a Euclidean distance of *ϵ* from *p*_*n*_. The set of points within the distance *ϵ* of *p*_*n*_ are considered to be in the neighbourhood of *p*_*n*_. Hence, the number of clusters and the number of pictures associated with any cluster depend on the size *ϵ* of the neighbourhood and on the minimum number of points *M* that must be present within each neighbourhood. We set *M* to 2% of the number of photos uploaded by any given user, and *ϵ*=0.5. This value is expressed in coordinate units, and sets the area of each neighbourhood at a value approximately equal to that of the Greater London metropolitan area.

**Figure 1.**
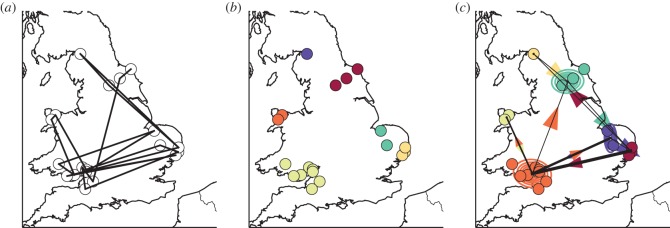
Model of an individual's mobility. (*a*) Individual trajectory depicting the location of geo-tagged photos uploaded by one of the users in the *Flickr* database. (*b*) Different colours indicate clusters discovered by the DBSCAN algorithm. (*c*) Different colours identify hidden states learned by a Hidden Markov Model, while the contour plots indicate Gaussian distributions learned for each state. The thickness of lines between different clusters is proportional to the number of times the user has moved between the two states, as estimated by the Viterbi algorithm. Arrows indicate the relative proportion of incoming and outgoing movements from one hidden state to the other.

Figure 1*a* shows the set of locations and the trajectory obtained from geo-tagged photos uploaded by a user, and figure 1*b* displays the result of clustering. Six distinct clusters are identified by the DBSCAN algorithm and are located, from south-west clockwise, around Bristol, northern Wales, Glasgow, North York Moors National Park, Norfolk and Suffolk.

We propose that the movement of *Flickr* users, observed by collecting geo-tagged metadata, is driven by unobserved factors. For example, one individual might reside in Bristol, visit relatives in Suffolk, and spend holidays in northern Wales. To model these high-level driving factors, we employ a Hidden Markov Model (HMM) [34]. HMMs are statistical models that comprise hidden states and emission probability distributions. Continuing with our example, the hidden state might take one of four values in the set {‘Home in Bristol’, ‘Family visit in Suffolk’, ‘Holiday in Wales’}. Each of the hidden states will generate observations according to a probability distribution conditional on the hidden state. In this study, we employ Gaussian emissions, which means that given a state, the observed location of geo-tagged photos emitted by that state follows a Gaussian probability distribution. Fitting the model to the observed data produces estimates of the parameters of the Gaussian distributions associated with each state, and of the transition probability describing how likely a user is to switch from one state to another. We infer a sequence of most likely states by using the Viterbi algorithm [35], which yields a trajectory between hidden states where each photo together with its corresponding time stamp is associated with a particular state.

We initialized the HMM model by setting a number of hidden states equal to the number of clusters identified by the DBSCAN algorithm, and by using the coordinates of the centroid of each cluster as the initial mean value of the corresponding Gaussian emission. Figure 1*c* depicts the model learned on the data depicted in figure 1*a*. A number of observations can be drawn from this model: firstly, we see that the clusters identified by the DBSCAN algorithm have been retained by the HMM. This is not guaranteed to be true in general, as DBSCAN only takes into account the spatial distribution of geo-tagged photos, whereas the HMM also incorporates information about the sequence of visited places that might determine a different mapping between locations and hidden states. The contour plots in figure 1*c* represent the Gaussian distributions learned for each hidden state, and the thickness of the lines connecting any two hidden states is proportional to the number of transitions between states, as estimated by the Viterbi algorithm. We note that the transition matrix is sparse, meaning that large movements only occur between some hidden states. This is also evident from figure 1*a*, as no movements are registered, for example, between northern Wales and Glasgow. We recognize the area around Bristol as the main source and destination of transitions, which suggests that this particular person might live there. Finally, the arrows in figure 1*c* depict the relative volume of incoming and outgoing transitions between any pair of clusters, hinting at the fact that there might be preferred travel sequences (for example, going from Bristol, to North York Moors National Park and Suffolk, but not in the opposite order).

### 2.2 Aggregate mobility model

**Figure 2.**
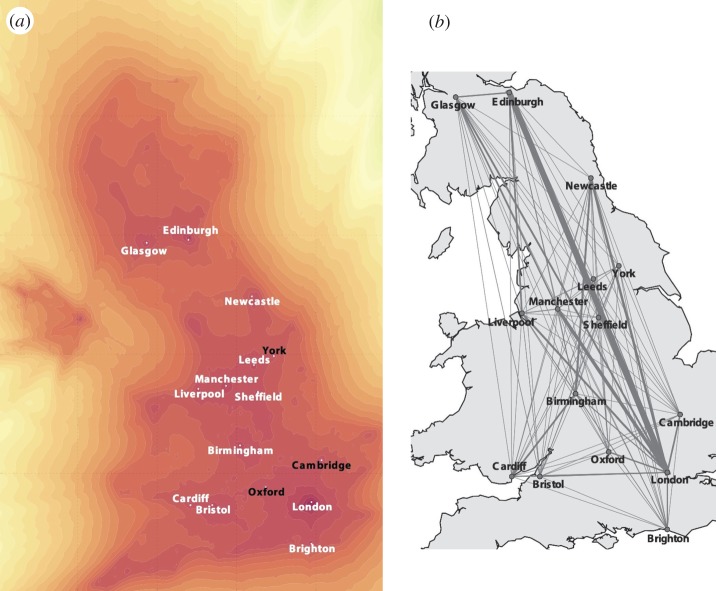
Aggregate model of mobility. (*a*) Probability of an individual's location derived from data uploaded by all the users in the *Flickr* dataset. The plot depicts the natural logarithm of *p*(***x***) as defined in equation (4.1). This describes the likelihood of finding a *Flickr* user in each geographical location and, since the dataset contains photos uploaded in the UK, it resembles the shape of the UK. The points in the map are local maxima identified with a maximum filter and thresholding, and correspond to the location of main UK cities. The names indicated in black indicate cities that do not appear in the list of the 20 most populous UK cities. (*b*) Aggregate transition probability between pairs of main UK cities. The line widths are proportional to *p*(***x***_d_,***x***_o_)+*p*(***x***_o_,***x***_d_) as defined in equation (4.2) and represent the probability of observing a transition between any two pairs of cities, aggregated over all the users in the dataset.

Having analysed the trajectory of a single user, we now focus on deriving aggregate results for all the users in the dataset. This will allow us to infer general patterns that describe the probability of finding any *Flickr* user in a given geographical area, and the probability of transition between pairs of areas. Figure 2*a* displays the function log⁡p( x) derived in equation (4.1) that describes the log-likelihood of finding a *Flickr* user in a given geographical location. The silhouette of Great Britain and Northern Ireland are clearly visible, along with areas of high probability corresponding to main UK cities. To obtain a set of points corresponding to maximum values of the function *p*(***x***), we employ a maximum filter. This is a commonly used tool in image processing that operates on a two-dimensional function by applying a sliding rectangular window of dimensions (*d*_*x*_,*d*_*y*_), selecting the local maximum within each window, and setting to zero all the other values. Since some areas do not contain notable local maxima (for example, regions located in open sea), we also thresholded the local maxima retaining only the ones with probability greater than a level *ϕ*.

**Figure 3.**
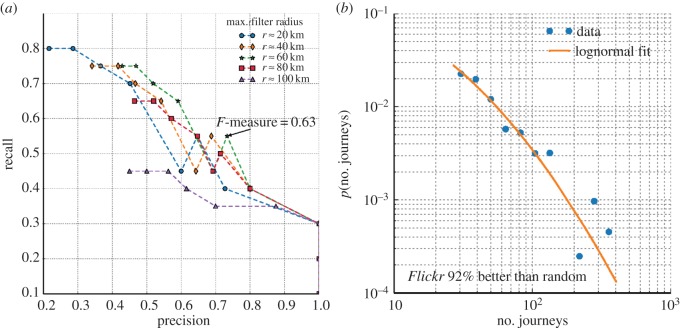
Evaluation of the aggregate model of mobility. (*a*) Identification of the 20 most populous UK cities. The plot depicts a tradeoff between precision (the number of correct identifications divided by the total number of cities identified) and recall (the number of correct identifications divided by 20) when a maximum filter is applied to the function *p*(***x***). Different lines correspond to different radii of the maximum filter, and values along the lines are obtained by varying the threshold *ϕ* between 10^−3^ and 10^−4^. The optimal parameters for the maximum filter corresponding to an *F*-measure of 0.63 are *r*≈60 km and *ϕ*=2.78×10^−3^. (*b*) The number of journeys per city pair as estimated from the NTS data is consistent with a lognormal distribution, with most pairs corresponding to less than 100 observed journeys, and a few pairs with at most about 400 journeys. We generated random variables according to this lognormal distribution, and computed their distance to the NTS estimates. We find that 92 out of 100 trials, the distance between the *Flickr* and NTS estimates is smaller than the distance between the randomly generated estimates and the NTS estimates.

We obtained a list of the 20 largest UK cities by number of resident population along with their geographical coordinates from Wikipedia (see the electronic supplementary material) to assess quantitatively whether the local maxima in figure 2*a* correspond to areas of large population. By varying the dimension of the maximum filter window and the threshold level, a different number of local maxima can be identified, hence determining a trade-off between precision (the number of correctly identified cities divided by the total number of maxima identified) and recall (the number of correctly identified cities divided by 20) of the cities' identification. For every local maximum computed on the function *p*(***x***), the point was judged to identify one of the cities in the list if it was located at a distance smaller than 15 km from the centre of the corresponding city, as computed by comparing the coordinates obtained from Wikipedia and the coordinates of the local maxima. Figure 3*a* depicts the tradeoff between precision and recall obtained by varying the sizes (*d*_*x*_,*d*_*y*_) between (18 km,28 km) and (90 km,140 km) expressed in terms of the radius $r=\sqrt{d_{x}d_{y}}$, and the threshold *ϕ* between 10^−3^ and 10^−4^. The *F*-measure is defined as the harmonic mean between precision and recall, and is a measure of the overall accuracy of the cities' identification. The maximum *F*-measure obtained was 0.63, corresponding to a size of (54 km,84 km) and a threshold *ϕ*=2.78×10^−3^. Figure 2*a* indicates the cities identified with these parameters, highlighting the ones that appear on the list of 20 most populous UK cities.

From the set of HMM models learned from all the users in our dataset, we can infer the probability that a user travels from any pair of geographical locations, hence obtaining a map of travel volumes between origin and destination pairs. Figure 2*b* depicts the aggregated transition probabilities between the cities corresponding to local maxima identified in figure 2*a*. For any pair of cities (***x***_d_,***x***_o_), the function *p*(***x***_d_,***x***_o_) derived in equation (4.2) describes the probability of observing a transition between city ***x***_o_ and city ***x***_d_ across all *Flickr* users. If we assume that the sum *p*(***x***_d_,***x***_o_)+*p*(***x***_o_,***x***_d_) is a proxy for the mobility flow occurring in both directions between the pair, we obtain values proportional to the thickness of the lines connecting any two cities in figure 2*b*. The largest flux is registered between London and Edinburgh, while other main fluxes are estimated between the capital and other major cities, as well as between Edinburgh and Glasgow and Cardiff and Birmingham. In general, fluxes between two cities appear to be positively correlated to the cities' sizes and negatively correlated to their mutual distance, that is in agreement with previous research on gravity models of human mobility [36].

**Figure 4.**
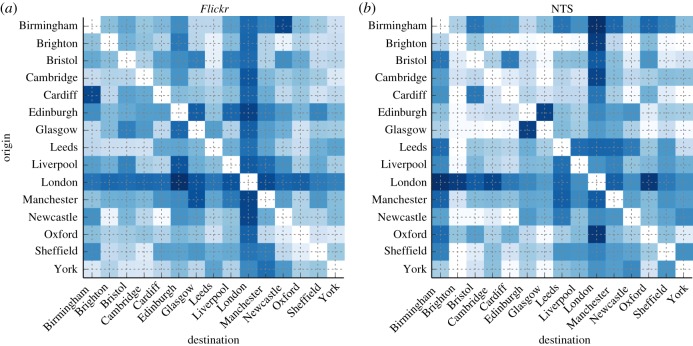
(*a*, *b*) Comparing *Flickr* estimates with official data. Number of journeys for each pair of cities of origin and destination as estimated by the model based on *Flickr* data and by the National Travel Survey (NTS) data. Each matrix displays values on a logarithmic scale from light blue (small number of journeys) to dark blue (large number of journeys). Units are not comparable between the two estimates, therefore the figures have been independently normalized in each matrix prior to visualization. The correlation between the two (non-normalized) estimates is moderate, but significant (Kendall's tau coefficient *τ*=0.45, *p*<0.001, *N*=210). Kendall's tau has been used instead of Pearson's correlation because the data are not normally distributed.

To assess whether these results are consistent with official statistics on mobility, we obtained a dataset from the National Travel Survey (NTS), the primary source of data on individuals' travel patterns in the UK (see the electronic supplementary material). NTS datasets are derived from annual surveys conducted with a sample of UK residents that report on various aspects of personal travel, such as means and reasons of journeys. Each record in the NTS database also specifies the origin and destination of journeys at the unitary authority boundary level (see the electronic supplementary material), that is an administrative entity which can be typically associated with a city in the UK. We selected data for the years from 2007 to 2013 (the same period covered by the *Flickr* dataset), counted the number of journeys for each origin/destination pair between the cities considered in figure 2*a*, and arranged the totals in a matrix of travel volumes across origin/destination pairs, as depicted in figure 4. For comparison, we also show the matrix of travel volumes obtained from the *Flickr* dataset. Some of the main trends are consistent across the two matrices, such as the prevalence of London as a source and destination of large travel volumes, and the large amount of journeys between Glasgow and Edinburgh. Other trends present in the NTS matrix, on the other hand, are not reflected by the data derived from *Flickr*, such as the very large travel volumes between London and nearby commuting cities like Brighton and Oxford.

In interpreting these results, two main factors regarding the NTS dataset need to be taken into account. On the one hand, unitary authority boundaries only loosely map to the areas covered by main UK cities, while our method uses the probability distribution learned from the *Flickr* dataset *p*(***x***_d_,***x***_o_), that we evaluated at the geographical coordinates associated with city centres. On the other hand, as we show in figure 3*b*, the amount of data present in the NTS dataset for most of the origin/destination pairs is very small for inferring general travel volumes (such statistics are not reported in the official document describing the survey's main findings). Nonetheless, we attempt a quantitative measure of the similarity between the *Flickr* and NTS origin/destination matrices by considering the distribution of the number of journeys reported by the NTS across origin/destination pairs. Figure 3*b* shows that this can be modelled using a lognormal distribution: journeys between most of the pairs are reported less than 100 times, while journeys between a small number of high volume origin/destination pairs are reported almost 400 times. We generate multiple random origin/destination matrices, sampling from a lognormal distribution whose parameters have been fitted to the NTS data. We compute the distance between the randomly generated matrices and the NTS matrix using the Frobenius distance, which corresponds to the Euclidean distance between vectors obtained by stacking all the values in each matrix along a vector. We find that in 92% of cases, the distance between the NTS matrix and the randomly generated matrices is greater than the distance between the NTS matrix and the *Flickr* matrix. This suggests that *Flickr* estimates reflect the main trends reported in the official data.

## 3. Conclusion

We propose a method for inferring in what geographical areas individuals are likely to be found, and between which areas they are likely to travel, by modelling data obtained from the photo-sharing website *Flickr*. Our approach is motivated by the finding that human mobility obeys universal statistical patterns mathematically described by Lévy flights, and that hidden or ‘latent’ factors that drive the emergence of these patterns can be modelled using machine learning techniques, yielding estimates of geographical location probabilities and transition probabilities between distant areas.

We aggregate models independently learned from the movements of about 16 000 *Flickr* users to infer general patterns of human mobility in the UK, essentially learning maps and travel flows from data alone. Although the evaluation of our method is sometimes difficult due to the lack of extensive official surveys on mobility at the country level, our findings appear to be in general agreement with the evidence available, providing a novel statistical tool for the analysis of online data sources, and adding to the evidence that online data can be used to quantify human travel.

The analysis presented here can also be extended by considering different spatio-temporal scales, such as movements between different city neighbourhoods, or by exploring seasonal patterns that may arise from individuals' trajectories or from aggregated travel patterns.

## 4. Material and methods

### 4.1 Data collection

We used the flickr.photo.search API to download metadata on all the publicly viewable photographs uploaded on the site between 2007 and 2013, for which both timestamps and geographical coordinates were available. The dataset was downloaded in March 2014. The resulting dataset consists of json records describing more than 140 million pictures uploaded by about 1.7 million users.

To compute the country in which each photograph was taken, we used country boundaries downloaded from Natural Earth Data in April 2014 (see the electronic supplementary material). Some photographs had coordinates which were associated with a sea location rather than a land location. These photographs were not associated with a country and were therefore removed from our analysis. We also removed users who only uploaded photographs on a single day. The data remaining for analysis describes photographs taken by roughly 1 million users. From this dataset, we selected photos taken in the UK, obtaining about 8 million photos uploaded by *ca* 16 thousand users.

Considering data from online photo-sharing platforms as a proxy for human mobility has inherent limitations, including the fact that pictures taken by a user a long time apart are likely to show an incomplete trajectory. However, this problem is mitigated by the fact that the time elapsed between consecutive photos follows a heavy tailed distribution (electronic supplementary material, figure S2), with most photos taken only a few hours or a few days apart, and only a small number of photos taken as much as a few years apart.

### 4.2 Aggregate model

The aggregate probability of finding a *Flickr* user in a geographical area ***x***=[*x*_lon_,*x*_lat_] defined by its longitude and latitude coordinates is given by the function:

4.1 $$p(\text{x})=\sum_{n=1}^{N}p(\text{x}\;|\;u_{n})p(u_{n}).$$

This is a sum of the probabilities *p*(***x*** | *u*_*n*_) which represent the likelihood of finding a given user *u*_*n*_ in the location ***x*** weighted by the probability that the observed data have been generated by user *u*_*n*_. The derivation of equation (4.1) from the *Flickr* dataset is detailed in the electronic supplementary material.

The probability of observing a transition between the origin coordinates ***x***_o_ and the destination coordinates ***x***_d_ is given by the function:

4.2 $$p({\text{x}}_{d},{\text{x}}_{o})=\sum_{n=1}^{N}p({\text{x}}_{d},{\text{x}}_{o}\;|\;u_{n})p(u_{n}).$$

This is a sum of the probabilities *p*(***x***_d_,***x***_o_ | *u*_*n*_) which represent the likelihood of observing the user *u*_*n*_ making a transition between the two geographical areas, weighted by *p*(*u*_*n*_) that indicates the likelihood of observing a transition generated by user *u*_*n*_. The derivation of equation (4.2) from the *Flickr* dataset is detailed in the electronic supplementary material.

## Supplementary Material

mobility.zip contains the following files that can be used to reproduce some of the figures in the manuscript (other figures need the NTS dataset that we cannot share, please see comments above) - displacements.csv: a list of displacement lengths to test Lévy flight behaviour - hmm_states: parameters of the HMM hidden states for each Flickr user - hmm_tmat.csv: transition matrix between hidden states for each user - marginals_prob.csv: marginal probability as a function of geographic location - marginals_tmat: transition probability between pairs of cities in the UK

## Data accessibility

A dataset containing metadata about the geo-tagged photos uploaded on *Flickr* is available as electronic supplementary material. This can be used to reproduce the results presented in this paper.
