# Supplementary material for: Modelling human mobility patterns using photographic data shared online
Source: R Soc Open Sci. 2015 Aug 12;2(8):150046. doi: 10.1098/rsos.150046 (PMC4555850; doi:10.1098/rsos.150046)
Supplement: mobility.zip contains the following files that can be used to reproduce some of the figures in the manuscript (other figures need the NTS dataset that we cannot share, please see comments above) - displacements.csv: a list of displacement lengths to test Lévy flight behaviour - hmm_states: parameter [file rsos150046supp1.pdf]

ROYAL SOCIETY OPEN SCIENCE

rsos.royalsocietypublishing.org

Research

Article submitted to journal

Subject Areas:
Complexity

Keywords:
computational social science, data science, social media, *Flickr*, human mobility, Lévy flights, complex systems

Modelling human mobility patterns using photographic data shared online (Supplementary Materials)

Daniele Barchiesi,[1*], Tobias Preis[1,2,3], Steven Bishop,[1] and Helen Susannah Moat[1,2,3]

[1]Department of Mathematics, UCL, Gower Street, London, WC1E 6BT, UK
[2]Warwick Business School, University of Warwick, Scarman Road, Coventry, CV4 7AL, UK
[3]Department of Physics, Boston University, 590 Commonwealth Avenue, Boston, MA 02215, USA

© 2014 The Authors. Published by the Royal Society under the terms of the Creative Commons Attribution License http://creativecommons.org/licenses/by/4.0/, which permits unrestricted use, provided the original author and source are credited.

THE ROYAL SOCIETY PUBLISHING

1. Retrieval of data

The following online resources have been used to retrieve data related to our study:

- Geographic coordinates of country boundaries [2].
- A list of the top 20 cities in the UK by population [1].
- A list of unitary authority boundaries in the UK [3].
- The National Travel Survey data detailing number of travels between unitary authority boundaries in the UK [4].

2. Distribution of displacement lengths

We can study the distribution of displacement lengths by analysing the difference between the coordinates of geo-tagged photos taken at consecutive times. Fig. 1 shows the empirical complementary cumulative distribution function (ccdf) computed from a random sample of 100000 displacements. This is a heavy tailed distribution, and is consistent with a Lévy flight behaviour. A random sample of the available data has been used to reduce the time needed to compute the ccdf. However, multiple trials on independent samples confirmed the results shown here.

3. Distribution of time intervals between consecutive photos

We can analyse the distribution of time intervals between photos by computing the time elapsed between the timestamps of any two photos uploaded by the same author at consecutive times. Fig. 2 shows the empirical complementary cumulative distribution function (ccdf) computed from a random sample of 100000 displacements. This is a heavy tailed distribution, and indicates that most photos are taken within intervals of a few days (e.g., the probability that the time elapsed between consecutive photos exceeds 3 days is about 0.1), but a few photos are taken years apart. A random sample of the available data has been used to reduce the time needed to compute the ccdf. However, multiple trials on independent samples confirmed the results shown here.

4. Derivation of marginal probability distributions

Let $\boldsymbol{x} = [x_{\mathsf{lon}}, x_{\mathsf{lat}}]$ be the longitude and latitude coordinates of a point. Given the output of a HMM, the probability $p(\boldsymbol{x}|u_n)$ of locating the user u_n in $\boldsymbol{x}$ is given by:

$$p(\boldsymbol{x}|u_n) = \sum_{i=1}^{S_n} p(\boldsymbol{x}|s_i, u_n) p(s_i|u_n)$$

where $p(\boldsymbol{x}|s_i, u_n)$ indicates the conditional probability distribution of the i-th Gaussian emission learned by the HMM for the user u_n, S_n is the total number of states estimated by the DBSCAN algorithm, and $p(s_i|u_n)$ is the prior probability of the state s_i conditional on the user u_n.

Let $\{h_{n,i}[m]\}$ indicate the set of photos uploaded by the user u_n that have been assigned by the Viterbi algorithm to the state s_i. We estimate the probability $p(s_i|u_n)$ by counting the number of these photos $M_{n,i} \stackrel{\text{def}}{=} |\{h_{n,i}[m]\}|$, and dividing it by the total number of photos uploaded by the user:

$$p(s_i|u_n) = \frac{M_{n,i}}{M_n}. \quad (4.1)$$

Therefore, the model estimates that users are more likely to be found in the areas where they uploaded more photos.

By aggregating $p(\boldsymbol{x}|u_n)$ over the entire set of users, we can estimate a probability distribution that describes the likelihood of finding any user in a given area. This will be the marginal probability

distribution:

$$p(\boldsymbol{x}) = \sum_{n=1}^{N} p(\boldsymbol{x}|u_n)p(u_n) \tag{4.2}$$

where N is the total number of users in the database, and an equal probability $p(u_n) = 1/N$ is assigned to every user.

Let us consider a single user u_n (whenever unambiguous from the context, we will avoid conditioning probabilities on u_n for clarity of notation). The parameters learned by the HMM describe the probability $p(\boldsymbol{x}|s)$ of finding the user in a particular location $\boldsymbol{x}$ given the value of the hidden state s, that corresponds to one of the clusters learned by the model, along with the transition probability $p(s_i|s_j)$ between states s_i and s_j.

A travel is defined by a pair of locations $\boldsymbol{x}_o$ and $\boldsymbol{x}_d$ that represent the origin and destination positions respectively. We are interested in the travel probability $p(\boldsymbol{x}_d, \boldsymbol{x}_o)$ that quantifies the likelihood of finding the user in locations $\boldsymbol{x}_d$ and $\boldsymbol{x}_o$ at consecutive times. This depends on the latent variables s_d and s_o.

$$\begin{aligned} p(\boldsymbol{x}_d, \boldsymbol{x}_o) &= \sum_{s_d \in S} \sum_{s_o \in S} p(\boldsymbol{x}_d, \boldsymbol{x}_o | s_d, s_o) p(s_d, s_o) \\ &= \sum_{s_d \in S} \sum_{s_o \in S} p(\boldsymbol{x}_d, \boldsymbol{x}_o | s_d, s_o) p(s_d|s_o) p(s_o) \end{aligned}$$

where S is the set of latent states learned for the user u_n. We assume that the variables $\boldsymbol{x}_d$ and $\boldsymbol{x}_o$ are independent conditional on the hidden states. Therefore, we can write the travel probability as:

$$p(\boldsymbol{x}_d, \boldsymbol{x}_o) = \sum_{s_d \in S} p(\boldsymbol{x}_d|s_d) \sum_{s_o \in S} p(\boldsymbol{x}_o|s_o) p(s_d|s_o) p(s_o). \tag{4.3}$$

The probability $p(s_o)$ can be derived from the Viterbi path, and has already been calculated in Eq. (5.1) as $p(s_i|u_n)$. Eq. (5.3) quantifies the likelihood that the user u_n travelled from $\boldsymbol{x}_o$ to $\boldsymbol{x}_d$, while the aggregate travel likelihood can be obtained as the marginal probability:

$$p(\boldsymbol{x}_d, \boldsymbol{x}_o) = \sum_{n} p(\boldsymbol{x}_d, \boldsymbol{x}_o | u_n) p(u_n) \tag{4.4}$$

where $p(u_n)$ describes the probability that we are observing the user u_n travelling between $\boldsymbol{x}_o$ and $\boldsymbol{x}_d$. We can estimate this quantity by counting the number of transitions between different latent states in the Viterbi path of the user $T_n = |\left\{h_n[m] : h_n[m+1] \in s_i, h_n[m] \in s_j, i \neq j\right\}|$, and dividing it by the total number of transitions estimated for all users:

$$p(u_n) = \frac{T_n}{\sum_n T_n}. \tag{4.5}$$

References

1. http://www.naturalearthdata.com/downloads/110m-cultural-vectors/.
2. http://en.wikipedia.org/wiki/List_of_urban_areas_in_the_United_Kingdom.
3. https://www.gov.uk/government/collections/national-travel-survey-statistics.
4. http://www.ons.gov.uk/ons/guide-method/geography/beginner-s-guide/administrative/england/counties/index.html.

5. Figures

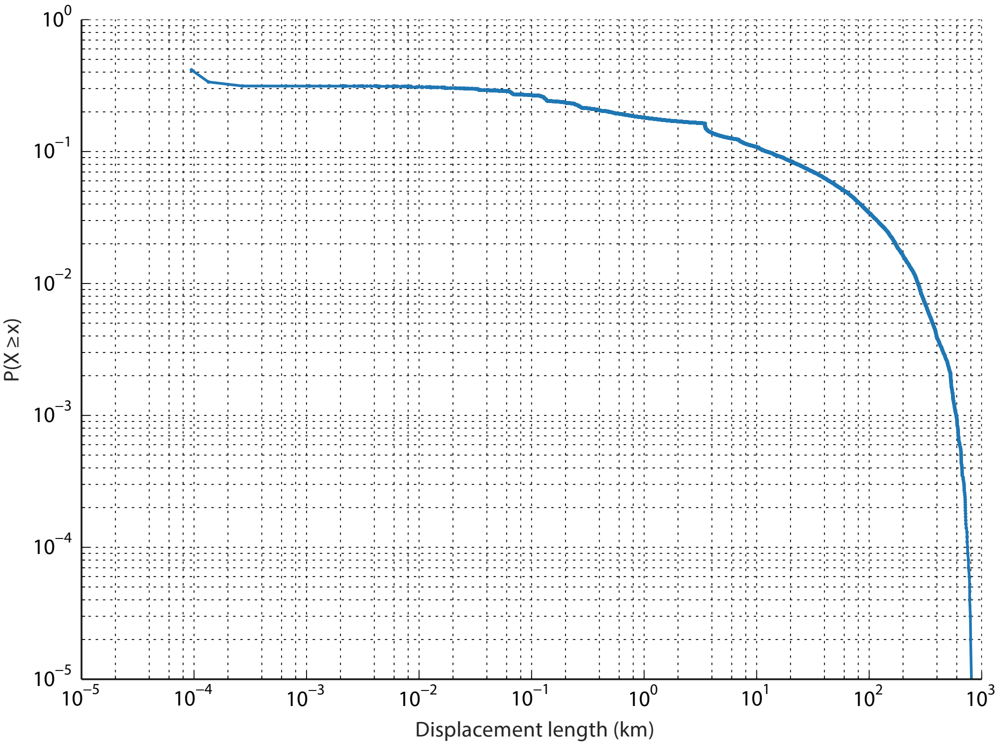

Figure 1: Tail distribution of displacements lengths. The plot depicts the complementary cumulative distribution function (ccdf) of displacement lengths in kilometres. The displacement lengths follow a heavy tailed distribution, that is consistent with a Lévy flight behaviour whereby a large number of small displacements is occasionally interspersed by large movements.

rsos.royalsocietypublishing.org R. Soc. open sci. 0000000

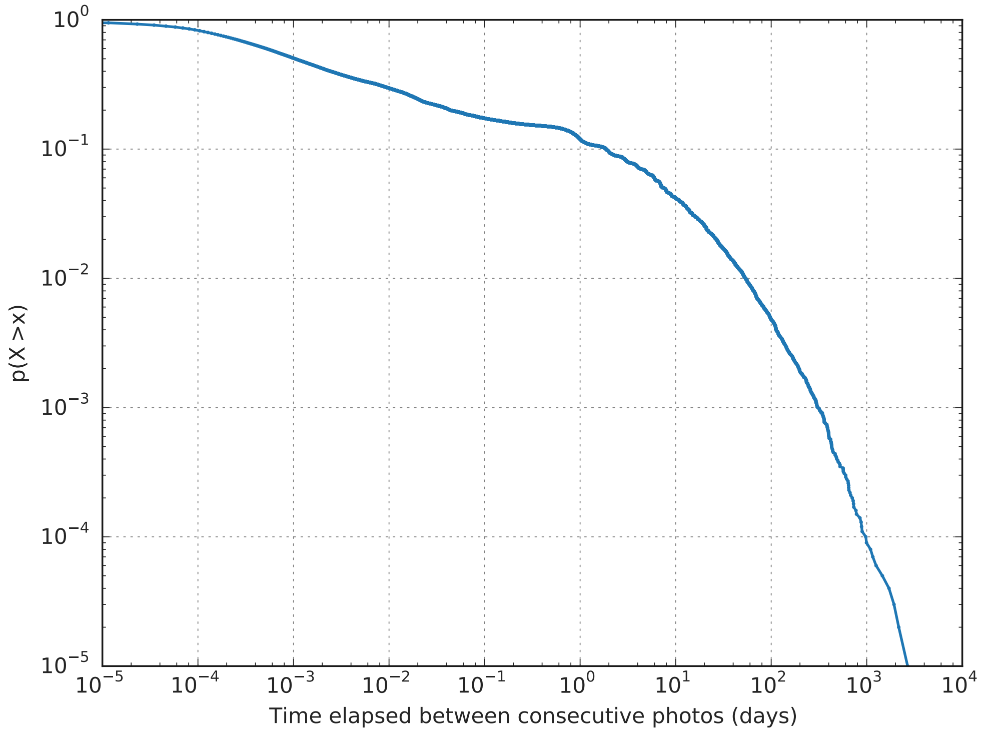

Figure 2: Tail distribution of time elapsed between consecutive photos. The plot depicts the complementary cumulative distribution function (ccdf) of the time elapsed between consecutive photos uploaded by a user. The time elapsed follows a heavy tailed distribution, which means that most photos are taken in the same day or only a few days apart, while some photos are taken as much as a few years apart.

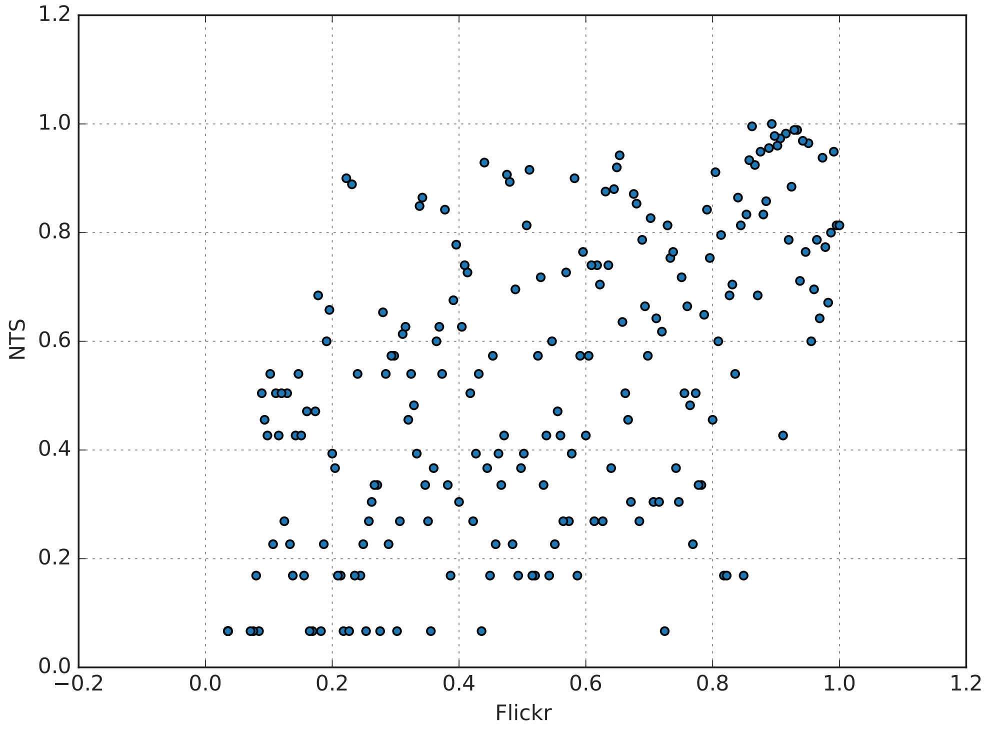

Figure 3: Correlation between the percentile rank of the number of travels reported in the NTS survey between pairs of major UK cities and the percentile rank of the probability of travel between the same cities, as learned by the HMM model. There is a moderate but significant correlation between the two variables (Kendall's tau coefficient $\tau = 0.45$, $p < 0.001$, $N = 210$). Kendall's tau has been used instead of Pearson's correlation because the data is not normally distributed.
